# Repeated Episodes of Ischemia/Reperfusion Induce Heme-Oxygenase-1 (HO-1) and Anti-Inflammatory Responses and Protects against Chronic Kidney Disease

**DOI:** 10.3390/ijms232314573

**Published:** 2022-11-23

**Authors:** Juan Antonio Ortega-Trejo, Rosalba Pérez-Villalva, Andrea Sánchez-Navarro, Brenda Marquina, Bernardo Rodríguez-Iturbe, Norma A. Bobadilla

**Affiliations:** 1 Molecular Physiology Unit, Instituto de Investigaciones Biomédicas, Universidad Nacional Autónoma de México, Mexico City 04510, Mexico; 2 Department of Nephrology and Mineral Metabolism, Instituto Nacional de Ciencias Médicas y Nutrición, Salvador Zubirán, Mexico City 14080, Mexico; 3 Department of Pathology, Instituto Nacional de Ciencias Médicas y Nutrición, Salvador Zubirán, Mexico City 14080, Mexico

**Keywords:** ischemic preconditioning, NFkB-p65, pro-inflammatory cytokines, renal fibrosis, AKI to CKD transition

## Abstract

Preconditioning episodes of ischemia/reperfusion (IR) induce protection against acute kidney injury (AKI), however their long-term effect still unknown. We evaluated AKI to chronic kidney disease (CKD) transition, after three-mild or three-severe episodes of IR. AKI was induced by single bilateral IR (1IR), or three episodes of IR separated by 10-day intervals (3IR) of mild (20 min) or severe (45 min) ischemia. Sham-operated rats served as controls. During 9-months, the 1IR group (20 or 45 min) developed CKD evidenced by progressive proteinuria and renal fibrosis. In contrast, the long-term adverse effects of AKI were markedly ameliorated in the 3IR group. The acute response in 3IR, contrasted with the 1IR group, that was characterized by an increment in heme oxygenase-1 (HO-1) and an anti-inflammatory response mediated by a NFkB-p65 phosphorylation and IL-6 decrease, together with an increase in TGF-β, and IL-10 expression, as well as in M2-macrophages. In addition, three episodes of IR downregulated endoplasmic reticulum (ER) stress markers expression, CHOP and BiP. Thus, repeated episodes of IR with 10-day intervals induced long-term renal protection accompanied with HO-1 overexpression and M2-macrophages increase.

## 1. Introduction

Chronic kidney disease (CKD) is a worldwide health problem. Estimates by the Global Burden of Disease study group shows that 1.2 million people died from CKD in 2017 and mortality associated to CKD increased 41.5% between 1990 to 2017 [1]. Accumulated experimental and clinical evidence has shown that acute kidney injury (AKI) represents one of the risk factors for the development of CKD [2,3,4,5,6,7,8,9,10].

AKI is a common syndrome that occurs in 21.6% of hospitalized patients and in nearly 50% in critically ill patients [11]. A cascade of events occurs during an AKI episode, including increased oxidative stress, mitochondrial dysfunction, brush border loss, necrosis, and apoptosis of proximal tubular epithelial cells [12,13]. Although the tubular epithelium has the capacity for regeneration, a maladaptive response may take place driving long-term tubulointerstitial fibrosis [14,15].

Recent studies have shown that AKI survivors are susceptible to further AKI episodes, an increased incidence of CKD, and increased mortality [16,17,18,19,20,21]. However, the associated comorbidities and the superimposed complications make difficult to define the risk of CKD associated with repeated episodes of AKI.

Studies of the protective effect induced by ischemic preconditioning have been reviewed by Wever, KE, et al. [22] and experimental studies have addressed the impact of repeated AKI episodes on renal injury [5,23,24,25,26,27]. Most, but not all these studies found improvement by ischemia preconditioning. Park et al. [25] showed that the benefits of 30 min of bilateral renal ischemia persists for 12 weeks and was associated with a significant increase in heat shock protein 25 (HSP25), endothelial (eNOS), and inducible nitric oxide synthase (iNOS) expression. Cho. et al. [23] demonstrated that a second episode of IR induced after one week improved renal function and was associated with lesser infiltration of inflammatory cells, increased IL-10 and reduced IL-6 expression. However, the notion that repeated episodes of AKI confer protection is contrary to the clinical observations. Indeed, Dong et al. [5] found that mice that underwent two insults of unilateral renal ischemia for 30 min and spaced by one week, showed worse renal function and fibrosis.

To our knowledge, the role of multiple episodes and the impact of the severity of IR, in relation to the transition to CKD have not been explored in long-term studies. Therefore, the aim of this study was to determine the impact of multiple mild or severe AKI episodes in the transition to CKD compared to a unique AKI episode. For reach this, we compared the effects of a single (1IR) or three episodes separated by 10-day intervals (3IR) of mild and severe bilateral kidney ischemia in groups of rats followed for 9 months. Because we found that repeated ischemic insults protect from the long-term CKD, we evaluated in another set of rats, the early renoprotective mechanisms developed because of ischemic preconditioning.

## 2. Results

**Figure 1:**
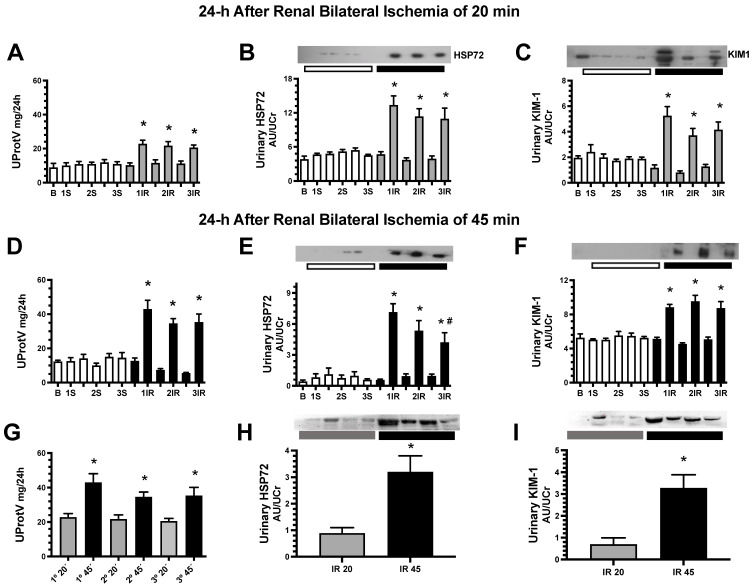
Proteinuria and urinary renal injury biomarkers measured before and 24 h after every AKI episode. In (**A**–**C**) are the results obtained from a mild and in (**D**,**E**) from a severe ischemic insult. (**A**,**D**,**G**) Urinary protein excretion, (**B**,**E**,**H**) urinary HSP72 and (**C**,**F**,**I**) urinary KIM1 assessed by WB analysis and corrected by urinary creatinine. Sham groups are represented in white bars, IR 20 min groups in gray bars and IR 45 min groups in black bars. Evaluations were performed at baseline (**B**), 24 h after each surgery (1S, 2S, 3S or 1IR, 2IR, 3IR) and two days before subsequent surgery (bars between surgeries). In (**G**,**H**,**I**), the renal injury provoked by 20 or 45 min of renal ischemia are compared. *n* = 6 to 10 per group. * *p* < 0.05 vs. sham group; # *p* < 0.05 vs. 1IR.

Renal function was analyzed before and after each episode of IR. As we previously reported [28], animals with a single IR episode for 20 or 45 min showed a significant increase in proteinuria (Figure 1A,D, respectively). A similar renal dysfunction was observed with each IR episode in both: mild (20 min) and severe AKI (45 min of ischemia). Noteworthy, two days before the rats underwent a new ischemic episode, proteinuria was completely recovered (Figure 1A,D, respectively). Furthermore, when we evaluated the urinary biomarkers of kidney damage, such as HSP72 and KIM-1 that were normalized by urinary creatinine, a significant increase was observed in each insult provoked. Interestingly, the magnitude was similar in each AKI episode (Figure 1B,C for 20 min, and 1E–F for 45 min of ischemia, respectively). Considering that Western blot analysis is semiquantitative and each membrane analyzed develops different chemiluminescence, the densitometric analysis varies in each determination. For this reason, we performed the comparison of mild and severe IR injury in the same membrane analyzed as appears in Figure 1H,I. As expected, the longer the ischemia time, the higher the proteinuria and the presence of urinary markers of renal injury (Figure 1G–I).

**Figure 2:**
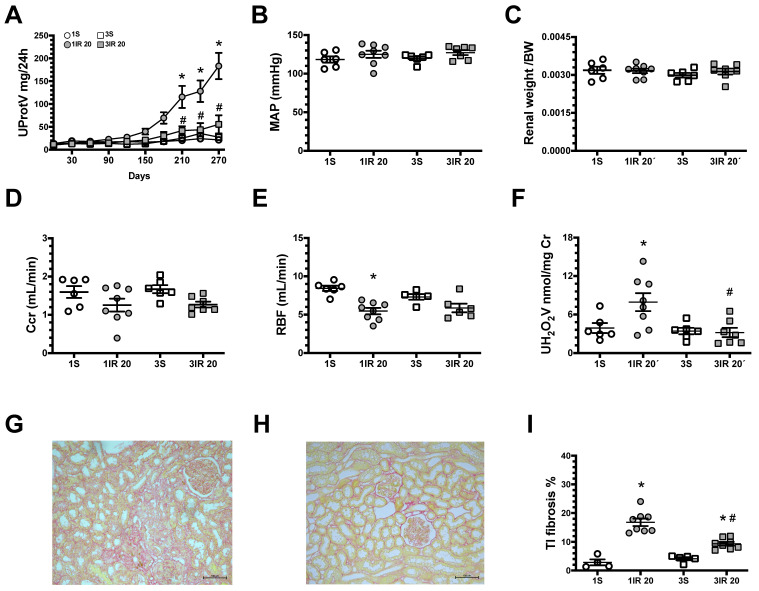
Repeated episodes of mild ischemic injury prevent the progression to CKD. (**A**) Urinary protein excretion measured every 30 days during nine months of the follow-up. At the end, the rats were studied: (**B**) mean arterial pressure (MAP), (**C**) renal weight/body weight ratio, (**D**) clearance of creatinine (Ccr), (**E**) renal blood flow (RBF), and (**F**) urinary H_2_O_2_ excretion. Representative microphotographs of Sirus red stained slices from a kidney rat underwent (**G**) one IR episode or (**H**) three IR episodes (magnification 150×), and in (**I**) tubule-interstitial fibrosis percentage. The sham group underwent one surgery (1S) is represented in white circles, rats underwent one IR episode for 20 min (1IR) in gray circles, sham group subjected to three false surgeries, one each 10 days (3S) is in white squares, and three episodes of 20 min, one each 10 days (3IR) is in gray squares. * *p* < 0.05 vs. both 1S or 3S; # *p* < 0.05 vs. 1IR group.

Long-term studies showed that the 1IR group had progressively increasing proteinuria (Figure 2A). In contrast, the 3IR group did not exhibit this gradual increase. After 9 months, the average of mean artery pressure (MAP), renal weight/body wight (BW), and creatinine clearance (Ccr) were similar among experimental groups (Figure 2B–D), but the 1IR group had had a reduction in renal blood flow (RBF) and greater oxidative stress, evaluated by urinary hydrogen peroxide excretion. The increment in oxidative stress was not observed in the 3IR group (Figure 2E,F). In association with these physiological and biochemical findings, a significant tubule-interstitial fibrosis (TI fibrosis) was observed in the 1IR group, and this structural injury was reduced in the 3IR group (Figure 2G–I). These results show that repeated mild ischemic insults delay the transition to CKD.

**Figure 3:**
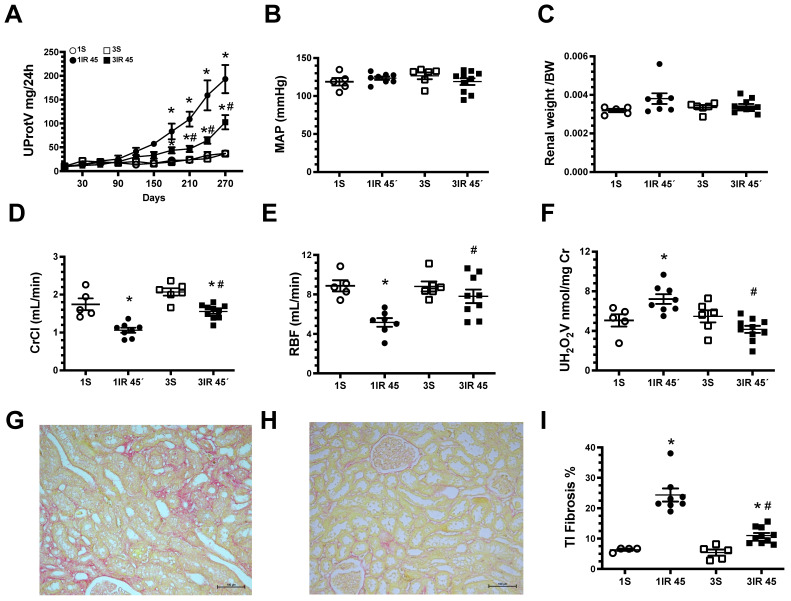
Repeated episodes of IR 45 min delayed the progression to chronic kidney disease. (**A**) Urinary protein excretion measured every 30 days during nine months of the follow-up. At the end, the rats were studied: (**B**) mean arterial pressure (MAP), (**C**) renal weight/body weight ratio, (**D**) creatinine clearance (Ccr), (**E**) renal blood flow (RBF), and (**F**) urinary H_2_O_2_ excretion. Representative microphotographs of Sirus red stained slices from a kidney rat underwent (**G**) one IR episode or (**H**) three IR episodes (magnification 150×), and in (**I**) tubule-interstitial fibrosis percentage. The sham group underwent one surgery (1S) is represented in white circles, rats underwent one IR episode for 45 min (1IR) in black circles, sham group subjected to three false surgeries, one each 10 days (3S) is in white squares, and three episodes of 45 min, one each 10 days (3IR) is in black squares. * *p* < 0.05 vs. both 1S or 3S; # *p* < 0.05 vs. 1IR group.

In Figure 3, are the results obtained when a severe insult was induced (45 min of ischemia). After 9 months, the 1IR group displayed evident CKD such as: proteinuria, renal dysfunction, increased oxidative stress, and greater TI fibrosis than 1IR group of 20 min. Although in the 3IR group exhibited proteinuria and renal fibrosis, the magnitude was significantly lower than the 1IR group. These results show that CKD progression after severe IR was ameliorated in the 3IR group, compared to the 1IR group.

The glomerular hypertrophy was evaluated by measuring the glomerular diameters that were distributed in seven ranges. The four control groups subjected to one or three sham surgeries had glomerular diameters in a bell shape distribution, in which more than 40% of glomeruli were in 100–124 μm (Figure S2A,C,E,G, respectively), as we have previously reported [2,6,29]. Whereas, both groups exposed to a single episode of ischemia for 20 or 45 min (Figure S2B,F) exhibited a significant increase in the proportion of glomeruli with a larger diameter (more than 125 μm, 60 and 80%, respectively). The distribution of glomerular diameters in the groups with three AKI episodes was like to the control groups (Figure S2D,H).

**Figure 4:**
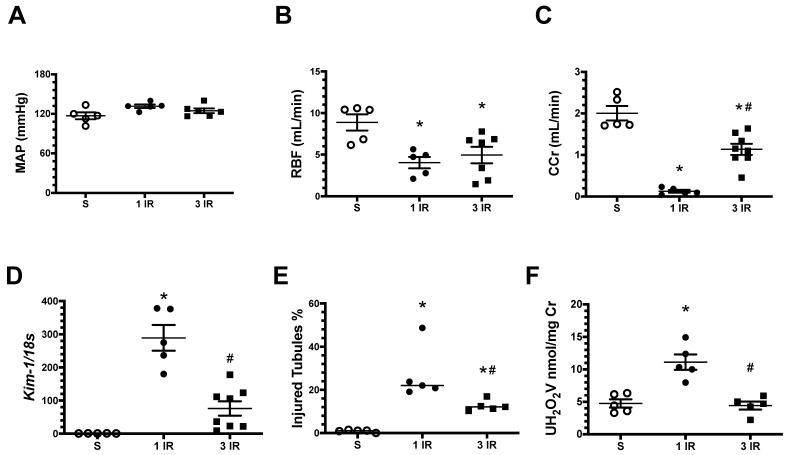
Repeated episodes of IR 45 min provoke renoprotection 24 h after the last episode. Animals with one or three episodes of IR were studied 24 h after the last episode. (**A**) Mean arterial pressure (MAP), (**B**) renal blood flow (RBF), (**C**) clearance of creatinine (Ccr), (**D**) Kim-1 mRNA levels, (**E**) percentage of injured tubules, and (**F**) urinary H_2_O_2_ excretion. The sham group is represented in white circles, the 1IR group in black circles, and the 3IR group in black squares. * *p* < 0.05 vs. S; # *p* < 0.05 vs. the 1IR group.

All these results, taken together show that the kidneys exposed to repeated AKI episodes develop a renoprotective response that reduce CKD progression. We then analyzed feasible pathways that were activated after the third episode of ischemic preconditioning in a separate set of experiments in rats that underwent 45 min of renal bilateral ischemia and were studied 24 h afterwards a single episode (1IR) or after a third episode (3IR) of IR. As is shown in Figure 4, the MAP was similar among the studied groups, and there was a similar reduction in RBF in the 1IR and in the 3IR groups. However, the 1IR group had a significant reduction in Ccr (Figure 4C), worse tubular damage (Figure 4D,E), and more oxidative stress (Figure 4F). These alterations were absent or ameliorated in the group 3IR group.

**Figure 5:**
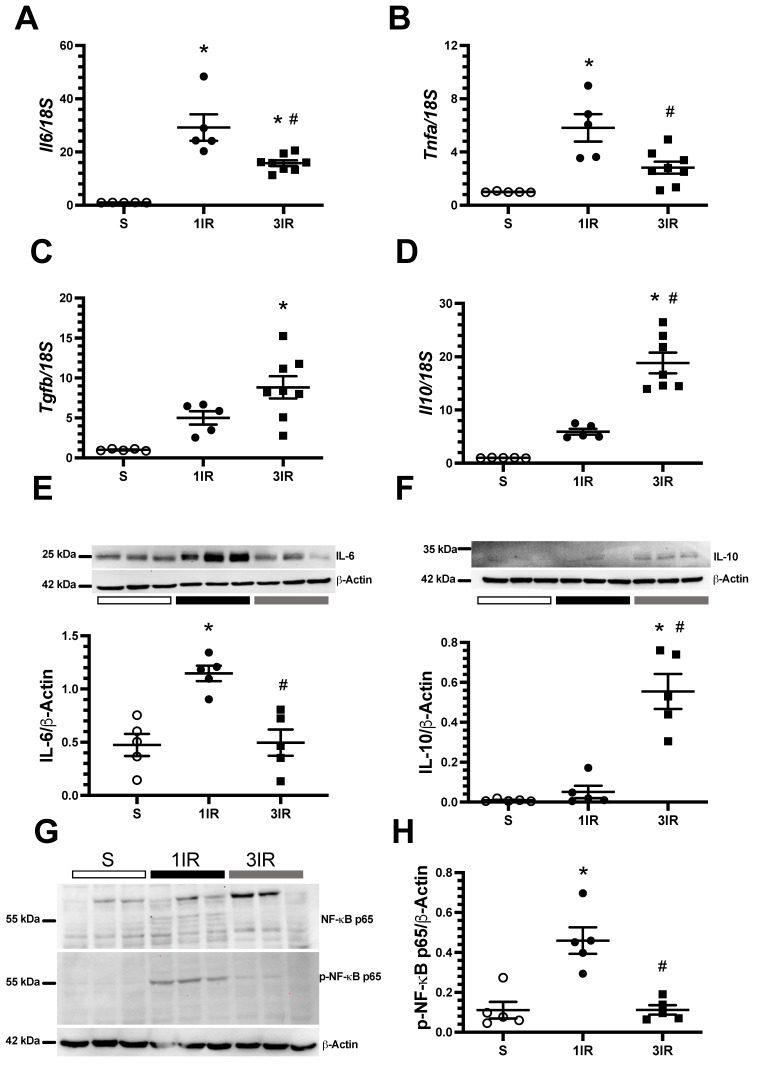
Three consecutive episodes of IR induced lesser renal inflammation than the 1IR group. (**A**) IL-6, (**B**) TNF-α, (**C**) TGF-β and (**D**) IL-10 mRNA levels. In (**E**) appears IL-6 protein and in (**F**) IL-10 protein expression, (**G**) representative images of Western blot analysis for NF-κB p65, p-NF-κB p65 (s536) and β-actin, and in (**H**) appears the ratio between p-NF-κB p65 and NF-κB p65. All these assessments were performed 24 h after the last IR episode. The sham group is represented in white circles, the 1IR group in black circles, and the 3IR group in black squares. * *p <* 0.05 vs. S; # *p* < 0.05 vs. 1IR group.

We further examined the expression of pro-inflammatory and anti-inflammatory mediators. The 1IR group exhibited a higher increase in the mRNA levels of pro-inflammatory cytokines *Il6* and *Tnfa* than the 3IR group (Figure 5A,B). In contrast, the mRNA levels of the anti-inflammatory cytokines *Tgfb* and *Il10* were only increased in the 3IR group (Figure 5C,D). We corroborated these results at protein level for IL-6 and IL-10 (Figure 5E,F). We assessed the protein levels of p65, a subunit of NF-kB and its phosphorylation, which is a critical in the regulation of genes that control inflammation [30]. Accordingly, with our findings on the pro-inflammatory cytokines profile, we found that 1IR group exhibited a significant elevation of p-NF-κB-p65, an effect that was not seen in the 3IR group (Figure 5G,H).

**Figure 6:**
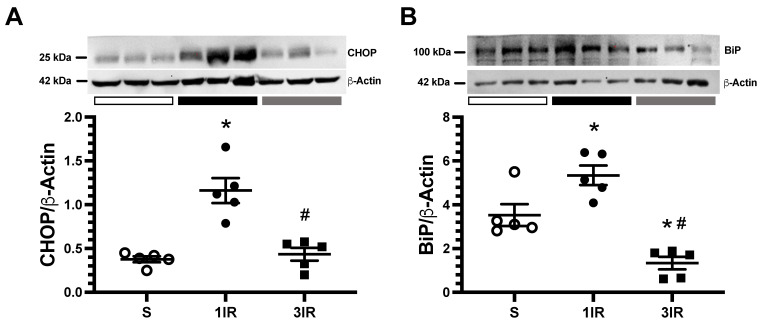
Repeated episodes of IR are associated with lesser endoplasmic reticulum stress. (**A**) CHOP and (**B**) BiP protein expression in renal cortex assessed by Western blot analysis. The sham group is represented in white circles, the 1IR group in black circles, and the 3IR group in black squares. * *p <* 0.05 vs. S, # *p* < 0.05 vs. 1IR group.

Additionally, the protein levels of CCAAT/enhancer-binding protein homologous protein (CHOP) and immunoglobulin binding protein (BiP) as endoplasmic reticulum stress markers were analyzed. CHOP and BiP expression were significantly up-regulated in the 1IR group. The 3IR group had similar CHOP expression and, interestingly, lower BiP expression than the control group (Figure 6A,B).

**Figure 7:**
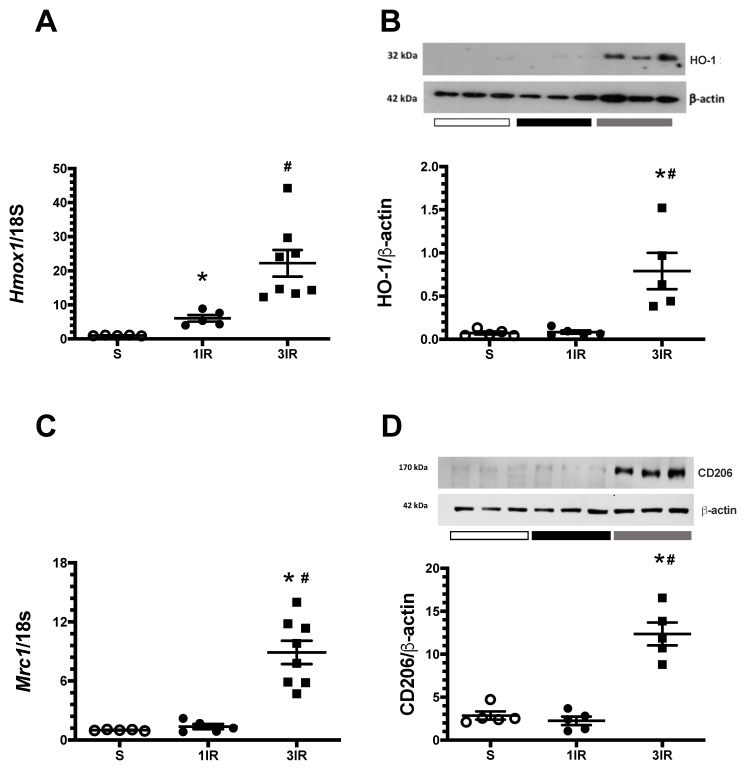
The renoprotective effect provoked by repeated AKI episodes was mediated by the induction of HO-1 and CD206 expression. (**A**) *Hmox1* mRNA levels, (**B**) HO-1 protein expression, (**C**) *Mrc1* mRNA levels and (**D**) CD206 protein expression, 24 h later that the last IR episode. The sham group is represented in white circles, the 1IR group in black circles, and the 3IR group in black squares. * *p* < 0.05 vs. S; # *p* < 0.05 vs. 1IR group.

To look for the possible mediators of renoprotection observed in the 3IR group, we examined the expression of the cytoprotective HSP32 chaperone, also known as heme oxygenase-1 (HO-1). In the 1IR group, *Hmox1* mRNA levels increased significantly (Figure 7A), but it was not reflected at protein level (Figure 7B). Interestingly, *Hmox1* increased by more than 20-fold at the mRNA level and by 5-fold at the protein level in the 3IR group as is depicted in Figure 7A,B, respectively. The expression of other chaperones was also analyzed. Figure S3 shows the kidney expression of HSP27, HSP72, and HSP90. The expression of HSP27 and HSP72 increased in both 1IR and 3IR groups, but only the HSP27 did not reach statistical difference in the 1IR group. After the ischemic injury, HSP90α and HSP90β expression did not change in the 1IR and 3IR groups. The differential expression between the 1IR and 3IR groups seen for HO-1, was not evident for other chaperons, suggesting that several ischemic insults drive a specific induction of HO-1 as a cytoprotector molecule as was previously reported [31,32].

Because a differential response in the pro and anti-inflammatory cytokines in the 1IR vs. 3IR groups was observed, we evaluated the mRNA and protein levels of CD206, also known as mannose receptor, that is a marker of macrophages type 2C typically immunomodulator with anti-inflammatory property. The 3IR group exhibited a remarkable increase in the expression of CD206 by 8-fold at the mRNA levels and by 4.3-fold at the protein level (Figure 7C,D, respectively). Such an effect was not seen after a single episode of IR.

**Figure 8:**
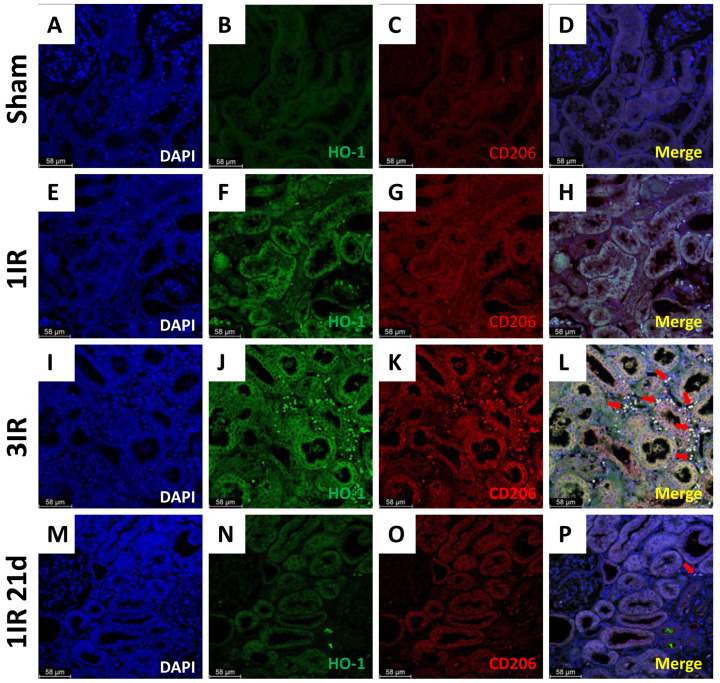
Immunofluorescence staining for HO-1 and CD206, a marker of M2c macrophage polarization in renal tissue. (**A**–**D**) Representative images of renal cortex from a sham rat, (**E**–**H**) from 1IR rat, (**I**–**L**) from 3IR rat, and (**M**–**P**) from 1IR rats just 21 days after the ischemic insult. DAPI is in blue color, HO-1 in green, and CD206 in red. The images stated as (**D**,**H**,**L**,**P**) show the corresponding merge images. Bar = 58 μm. Arrows in red show the co-immunolocalization of interstitial positive cells for HO-1 and CD206.

Furthermore, the immunostaining for HO-1 and CD206 are shown in Figure 8. The staining for HO-1 was marginal in the sham group (Figure 8B), compared with the ischemic groups. In the 1IR group, HO-1 was mainly found in the tubular epithelium (Figure 8F). In contrast, the 3IR group exhibited a large amount of HO-1 in the interstitial section in addition to its presence in tubular epithelium. The same finding was observed for the CD206 staining for the 3IR group (Figure 8K). There was a substantial colocalization of HO-1 and CD206 proteins, suggesting that the same cells are expressing both proteins (Figure 8L). Because the 3IR group was studied after 21d of their first surgery, we included another group with only one IR episode of 45 min but studied after 21d (1IR 21d). The 1IR 21d group did not show the elevation in the expression of HO-1 and CD206, as was observed in the 3IR group (Figure 8N,O).

## 3. Discussion

Previous studies performed with two-episodes of renal bilateral ischemia have found to be beneficial for the kidney [23] or resulting in accelerated AKI to CKD transition [5]. To our knowledge, this is the first study to identify the long-term kidney consequences resulting from single and multiple episodes of AKI of different severity. In this study, we found that a single episode of mild (20 min) or severe (45 min) renal bilateral ischemia induced increasing proteinuria that 9 months after IR reached levels about 200 mg/24 h. At this point, the rats exhibited a reduction in RBF and creatinine clearance and increased oxidative stress and tubulo-interstitial fibrosis were demonstrated. Even though, ischemic preconditioning with three episodes of IR, did not entirely prevented these alterations, offered a significant protection, evidenced by lower manifestations of kidney injury. Therefore, this study confirmed that CKD is a long-term consequence of AKI, which is in agreement with clinical observations [7,33,34], and in addition, that repeated episodes of IR offer a significant protection against subsequent CKD. This protective ischemic preconditioning was reported for the first time by Murry CE, et al. [35] in the myocardium. The protective effect of repeated IR episodes should not be interpreted to be against the prevalent clinical wisdom in nephrology that consecutive episodes of AKI increase the provability of progression to CKD. There is a limited interval of time in which ischemic preconditioning drives protective mechanisms. Most experimental studies have used intervals of hours or days and in the present study, the interval was of 10 days. In the clinical situation, repeated episodes of AKI are likely to take place at much longer and variable intervals and, therefore, ischemic preconditioning is unlikely to be activated. In addition, the comorbidities that are present in patients likely contribute to the long-term effects of episodes of AKI. The importance of comorbidities has been shown in diabetic rats, where preconditioning ischemic episodes aggravate the damage [36].

We investigated the potential causes of the long-term protection of repeated episodes of IR in studies done shortly after 1IR and 3IR episodes. It should be noted that the markers of kidney injury (proteinuria and urinary excretion of HSP72 and KIM-1) had returned to control levels in the 10th day interval between the episodes of IR (Figure 1). The beneficial effects of repeated AKI episodes are associated with reduced inflammation, as shown by reduced mRNA and protein expression of proinflammatory IL-6 and *tnfa,* and increased anti-inflammatory *tgfb* and IL-10 after the third IR episode (Figure 5). The activation of NF-κB is an important mechanism in the pathogenesis of AKI to CKD transition and it has been demonstrated that its suppression specifically in macrophages alleviates the renal damage [37]. Here we found that the phosphorylation of NF-κB p65 subunit was elevated in the 1IR group, but this post-translational modification was absent in the 3IR group, suggesting that an anti-inflammatory environment predominates in these rats.

Furthermore, markers of endoplasmic reticulum stress, CHOP and BiP, that were evident after one episode of IR were reduced after the third episode of IR. Interestingly, the 3IR group had BiP levels lower than the sham-operated group, suggesting that constitutive BiP levels are suppressed by repeated IR episodes (Figure 6).

The protective findings of repeated IR have been associated in previous studies with increased expression of heat shock proteins (HSPs) [38], induction of transcription factor Nrf2, which regulate antioxidant genes expression [39], increased iNOS [25], regulatory T cells in the kidney [23], and a decreased production of pro-inflammatory cytokines [40]. Protection derived from these factors have been assumed to result from reducing leukocyte-endothelial interactions and improving inflammation and oxidative stress. We were prompted to look into the role of heme oxygenase 1 (HO-1) because is an adaptive response that plays a central role in improving oxidative stress and inflammation which are key elements in IR [31,41,42]. HO is expressed in all cell types, with a variety of transcription factors such as AP-1, Nrf2, HIF-1 and HSF [43,44,45], and its canonical function is to degrade heme group to biliverdin with the release of equimolar amounts of carbon monoxide and Fe^2+^. Three isoforms of HO have been described: inducible HO-1 and constitutive HO-2 and HO-3 [46]. HO-1 induction stimuli are heat, oxidative stress, heavy metals, ultraviolet irradiation, and cytokines, like IL-10 [47]. In the present study, we found that repeated episodes of IR induced a strong HO-1 expression in the kidney (Figure 7) accompanied with an elevation in protein and mRNA levels of IL-10 (Figure 5). Also, it has been reported in silico a possible interaction between NF-κB p65 subunit and HO-1 [48] and in vitro studies have shown that HO-1 inhibits the nuclear translocation of NF-κB-p65 [49,50]. To be noted, Ferenbach, DA, et al. demonstrated that the induction of HO-1 with heme arginate protects against AKI in aging mice [51].

In our studies, the renoprotection observed in the 3IR group was associated with overexpression of HO-1 and CD206 (mannose receptor), suggesting that the axis IL-10/HO-1 in M2 macrophages plays a role in the protection resulting from three episodes of IR (Figure 7). Macrophage proliferation and differentiation from an M1 to M2 profile occurs within 3 to 5 days after an IR episode [52], and the HO-1 overexpression was found 24 h after the 3rd AKI episode. HO-1 is known to be expressed in the proximal tubule, but the basal levels are low [46]. In our studies we found that repeated IR induced HO-1 staining in tubulointerstitial sections with important co-localization with CD206 (Figure 8). This finding was not a delayed effect of IR, since it was not observed in studies done 21 days after a single episode of IR (Figure 8N,O). However, further studies are required to assess whether pharmacological induction of HO-1 could be sufficient to improve AKI to CKD transition.

The limitations of the present study are: (1) we evaluated only young and healthy rats therefore, a new field is open to evaluate the effect of preconditioning in rats with renal diseases such as obesity, diabetic nephropathy, hypertension, etc. These scenarios could resemble the situation that normally happen in the clinical setting, where the patients exhibited one or more co-morbidities. (2) The blockade of M2 polarization was not assessed in this study, to conclusively determine the influence of this type of cells in the renoprotection observed in the group exposed to multiple IR.

In summary, we show that renal IR induced the late development of CKD and that repeated ischemic episodes at 10-day intervals protected against AKI to CKD transition. A possible renoprotective mechanism involved is the induction of HO-1 that appears to be released by M2 macrophages, in addition to the fact that the improvement in early inflammatory kidney damage and lower ER stress are likely the reason for the subsequent amelioration of CKD. Our study highlights the potential use of renal preconditioning, which could even be remote, to prevent long-term consequences of AKI in the clinical setting such as organ transplantation or cardiovascular surgery.

## 4. Material and Methods

All experiments involving animals were conducted in strict accordance with the NIH *Guide for the Care and Use of Laboratory Animals* and with the Mexican Federal Regulation for animal reproduction, care, and experimentation (NOM-062-ZOO-2001). The study was approved by the Animal Care and Use Committee of the Instituto Nacional de Ciencias Médicas y Nutrición Salvador Zubirán (NMM-1893), Mexico City. All the animals were housed on a 12:12 h light-dark cycle at an average temperature of 21 °C and allowed free access to water and standard rat food.

### 4.1. Protocol 1

Fifty-four male Wistar rats weighting 280–300 g were used. Ischemic AKI was induced with mild (20 min) or severe (45 min) bilateral occlusion of the renal pedicles. Rats were randomly allocated to sham surgery (S) or ischemia/reperfusion (IR) in the groups that were given a single (S1, IR1) or three episodes (S3, IR3) of experimental intervention. Repeated IR episodes were induced at 10-day intervals. Two days after each surgery and every month afterward rats were placed in metabolic cages for 24 h urine collection. Animals were followed for nine months after the initial IR episode (Figure S1).

### 4.2. Protocol 2

Early changes after IR were studied in a separate set of experiments done in 18 male Wistar rats randomly assigned to three groups: Sham-operated (S), one episode of bilateral renal ischemia for 45 min (1IR), and three episodes of bilateral renal ischemia for 45 min, applied with an interval of ten days (3IR). 24 h after the single or the last surgery in the 3IR group, animals were placed in metabolic cages for 24 h urine collection and were euthanized afterwards.

### 4.3. Ischemia Reperfusion Model of Acute Kidney Injury

AKI was induced by bilateral renal ischemia. Briefly, general anesthesia was induced by an i.p. injection of sodium pentobarbital (30 mg/kg). Both kidneys were exposed through a midline abdominal incision and renal pedicles were clamped with non-traumatic clamps for 20 or 45 min as we previously reported [2,6,29,53]. Sham-operated rats had a similar surgical procedure (laparotomy and renal pedicle dissection) without clamping the kidney vessels.

### 4.4. Biochemical and Renal Hemodynamics

Urinary protein excretion was determined in 24 h urinary collection by the TCA turbidimetric method. At the end of the experimental protocols, rats were anesthetized with sodium pentobarbital (30 mg/kg) and placed on a homoeothermic table. Trachea and left femoral artery were cannulated with polyethylene tubing PE-240 and PE-50, respectively. The mean arterial pressure (MAP) was monitored with a pressure transducer (model p23 db, Gould) and recorded on a polygraph (Grass Instruments, Quincy, MA, USA). An ultrasound transit-time flow probe was placed around the left renal artery to register the renal blood flow (RBF). At the end of the experiment, blood samples were taken, and the left kidney was removed and separated by dissection into cortex and medulla, frozen in liquid nitrogen and stored at −80 °C. The right kidneys were perfused with physiological solution and with a freshly prepared 4% formalin buffer to fix the tissue. Serum and urine creatinine concentration were measured with the Quantichrom creatinine assay kit (DICT-500, Bioassay system) and the amount of hydrogen peroxide (H_2_O_2_) in urine was determined with an Amplex Red Hydrogen Peroxide/Peroxidase Assay Kit (Invitrogen) according to manufacturer’s instructions.

### 4.5. Histopathological Analysis

The right kidney was perfused until fixation was completed, maintaining the MAP that each rat had during the experiment. After appropriate dehydration, renal tissue was embedded in paraffin, sectioned at 3 μm and stained with periodic acid-Schiff (PAS) or Sirius red. Light microscopy microphotographies of at least, five subcortical fields were taken from each kidney slide using a digital camera incorporated in a Nikon microscope (magnification 200×). The glomerular diameter was measured as we have previously reported [2,6,29]. Tubulo-interstitial fibrosis evidenced by Sirus red stained was quantified using the software NIS elements and the percentage of tubulointerstitial fibrosis was calculated by dividing the fibrotic area by the total area. All histological studies were done by investigators blinded to the experimental group.

### 4.6. Immunofluorescence

Renal slices of 4 μm from tissue embedded in paraffin were deparaffined. The antibody recovery was made with citrate buffer (Cat. No. BSB 0022, Bio SB) for 12 min in high pressure. The slides were blocked for 20 min with a 10% bovine serum albumin (BSA) solution prepared with TBS 1X Tween 0.1% (pH 7.4; prepared freshly and stored at 4 °C for use within 48 h). Primary antibody HO-1 (1:200, Cat. No. sc-390991, Santa Cruz, Dallas, TX, USA), and CD206 (mannose receptor) (1:1000, Cat. No. ab64693, Abcam, Waltham, MA, USA) were applied to slides incubated in a humid chamber overnight at 4 °C. After 3 washes with TBS 1X Tween 0.1%, the slides were incubated with secondary antibodies anti-mouse Alexa Fluor-488 (1:1000, Cat. No. A32723, ThermoFisher, Waltham, MA, USA) and anti-rabbit Alexa Fluor-594 (1:1000, Cat. No. A32740, ThermoFisher) and protected from light 1 h at room temperature. After 3 washes, they were incubated with DAPI solution (1:20,000) for 10 min. Finally, the assembly was done with mounting solution and a coverslip was placed. The slides were cover from light until photos 40× were taken using a Leica DMi8 (Leica, Wetzlar, Germany).

### 4.7. Semiquantitative RT-PCR

Total RNA was isolated from the left kidney using the TRIzol method (Invitrogen, Carlsbad, CA, USA), and RNA integrity was confirmed by 1% agarose gel electrophoresis. Reverse transcription was conducted with 1 μg of total RNA and 200 U of Moloney murine leukemia virus reverse transcriptase (Invitrogen). The mRNA levels of *Havcr1* (Rn00597703_m1), *Il6* (Rn01410330_m1), *Tnfa* (Rn99999017_m1), *Tgfb* (Rn00572010_m1), *Il10* (Rn99999012_m1), *Hmox1* (Rn00561387_m1), *Mrc1* (Rn01487342_m1) were quantified by real-time PCR on a QuantStudio 5 (Life Technologies). Eukaryotic 18S rRNA was used as endogenous control (Predesigned assay reagent, external run, Rn03928990_g1, Cat. No. 4319413E). The relative expression was performed with the comparative threshold cycle (Ct) method [54].

### 4.8. Western Blot Analysis

Total renal proteins were isolated from renal cortex from each rat and homogenized in lysis buffer (50 mM HEPES pH 7.4, 250 mM NaCl, 5 mM EDTA, 0.1% NP-40, and complete protease inhibitor (Roche)). Protein samples was resolved by SDS-PAGE electrophoresis and electroblotted into polyvinylidinedifluoride (PVDF) membranes (Millipore). Membranes were then blocked with 5% blotting-grade non-fat dry milk. Membranes were then incubated in 5% blotting-grade non-fat dry milk with their respective antibodies. Specific antibodies against HSP72 (Enzo, ADI-SPA-810F), KIM-1 (Boster Biological tech, PA1632), CHOP (cell signaling, 2895), BiP (cell signaling, 3177), HO-1 (also named HSP32) (Santa Cruz, sc390991), CD206 (Abcam, ab64693), IL-6 (Santa Cruz, sc57315), IL-10 (Santa Cruz, sc-365858), HSP27 (Santa Cruz, sc51956), Hsp90α (Abcam, ab2928), Hsp90β (Abcam, ab2927), NF-κB p65 (Santa Cruz, sc-8008) and p-NF-κB p65 (s536) (Santa Cruz, sc-135858) were used. After overnight at 4 °C incubation with primary antibody, membranes were washed and incubated with their respective secondary antibody (Jackson, 115-035-174, anti-mouse, 1:1500 or Sigma, A0545, anti-rabbit, 1:5000). As a loading control, membranes were incubated overnight at 4 °C with anti-actin antibody conjugated to HRP (Abcam, ab49900 1:1,500,000 dilution). p-NF-κB p65 blots were stripped for 15 min with Re-blot Plus strong solution 10× (Millipore, 2504). Proteins were detected with an enhanced chemiluminescence kit (Millipore, Burlington, MA, USA). In the case of HSP72 and KIM-1 urinary excretion, the values of optical density were corrected by urinary creatinine, as we have previously reported [53,55].

## 5. Statistical Analysis

The results are presented as the mean ± Standard error (SE). The significance of the differences between the groups was assessed by analysis of variance (ANOVA) using the Bonferroni post-hoc test for multiple comparisons. Statistical analyses were performed using GraphPad Prism version 9 for Mac OS X (GraphPad Software, La Jolla, CA, USA). Statistical significance was defined as a two-tailed *p* value < 0.05.

## Data Availability

The data that support the findings of this study are available in the methods and/or Supplementary Material of this article.

## Supplementary Materials

The following supporting information can be downloaded at: https://www.mdpi.com/article/10.3390/ijms232314573/s1.
