# Supplementary material for: Repeated Episodes of Ischemia/Reperfusion Induce Heme-Oxygenase-1 (HO-1) and Anti-Inflammatory Responses and Protects against Chronic Kidney Disease
Source: Int J Mol Sci. 2022 Nov 23;23(23):14573. doi: 10.3390/ijms232314573 (PMC9739146; doi:10.3390/ijms232314573)
Supplement: Supplementary file 1 [file ijms-23-14573-s001.zip › ijms-1969665-supplementary.pdf]

Repeated episodes of ischemia/reperfusion induce hemoxigenase-1 (HO-1) and anti-inflammatory responses and protects against chronic kidney injury.

Juan Antonio Ortega-Trejo[1,2], Rosalba Pérez-Villalva[1,2], Andrea Sánchez-Navarro[1,2], Brenda Marquina[3], Bernardo Rodríguez-Iturbe[2] and Norma A. Bobadilla[1,2].

[1]Molecular Physiology Unit, Instituto de Investigaciones Biomédicas, Universidad Nacional Autónoma de México, [2]Departments of Nephrology and Mineral Metabolism and [3]Pathology Instituto Nacional de Ciencias Médicas y Nutrición, Salvador Zubirán, Mexico City, Mexico.

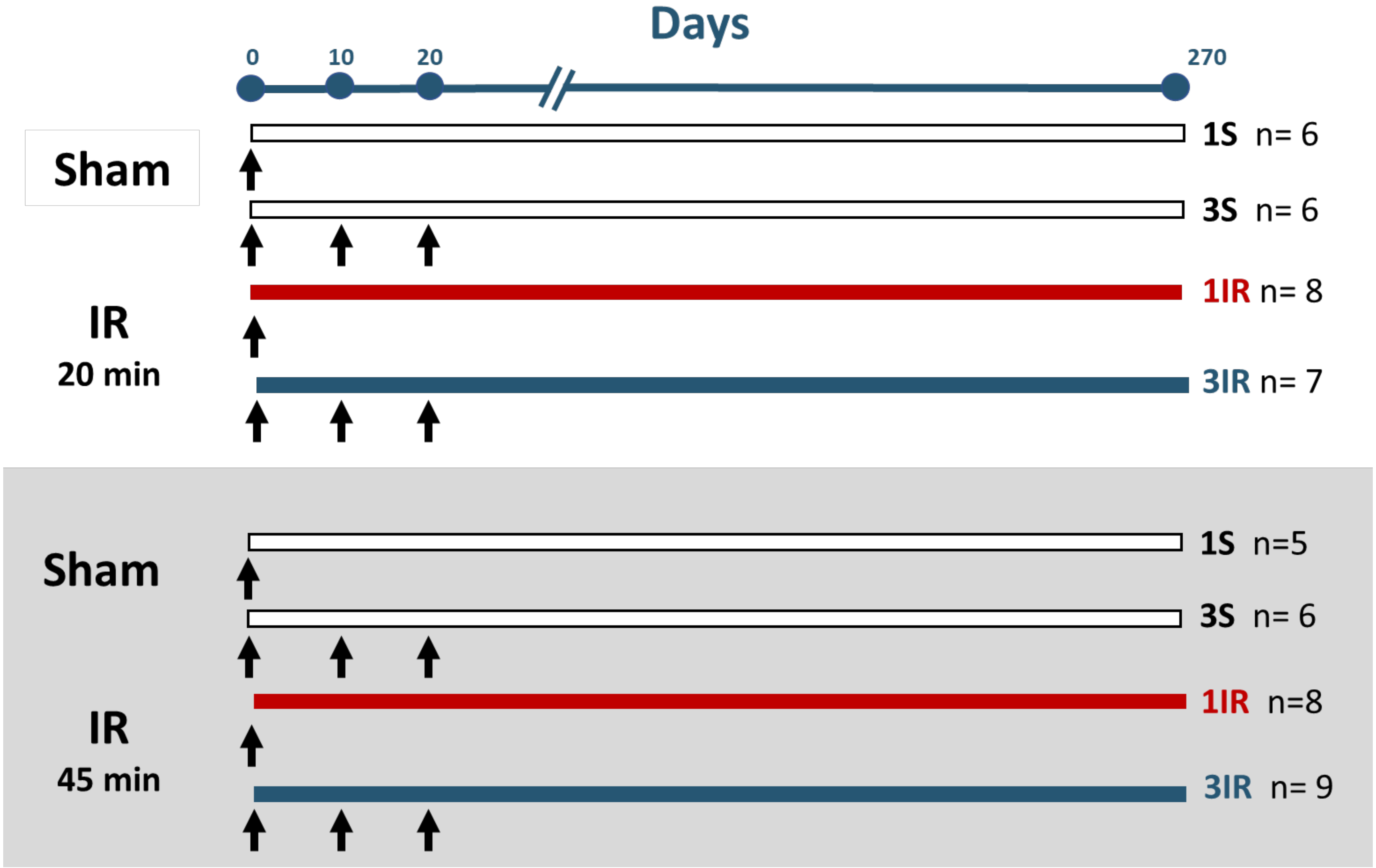

Suppl. Figure S1. Experimental Protocol. Acute kidney injury was induced by bilateral renal ischemia in male Wistar rats. A mild (20 min) or severe ischemic injury (45 min of ischemia) were studied. In each case two experimental groups were included, the group subjected two only one AKI episode (1IR) and the group underwent three AKI episodes (3IR) every ten days and compared with their respective sham operated groups. The animals were studied after 270 days. The number of rats that conformed each group is stated in the Figure.

Glomerular Diameter Distribution (Renal Ischemia of 20 min)

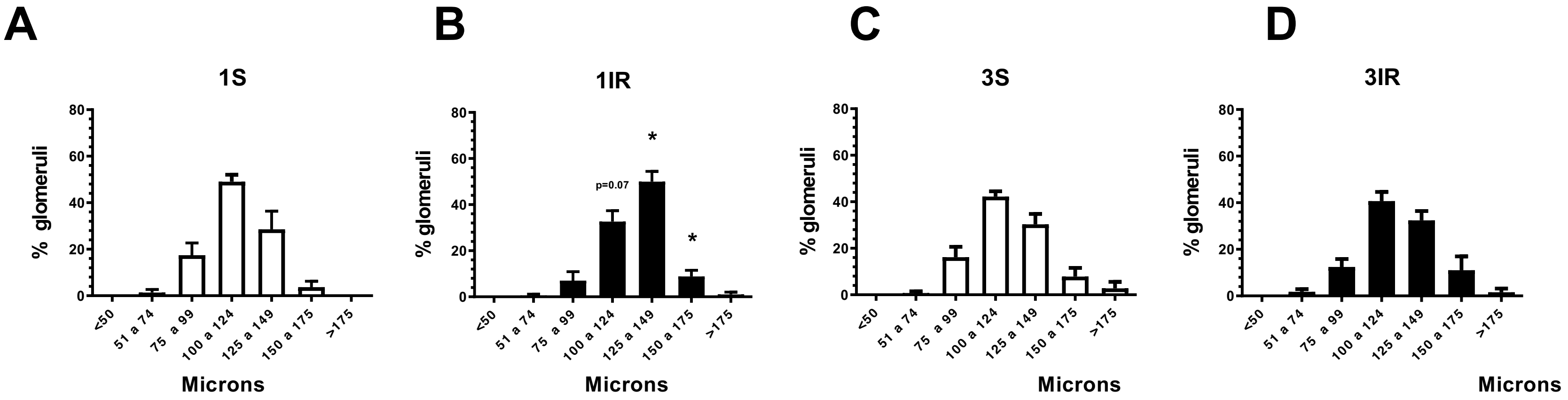

Glomerular Diameter Distribution (Renal Ischemia of 45 min)

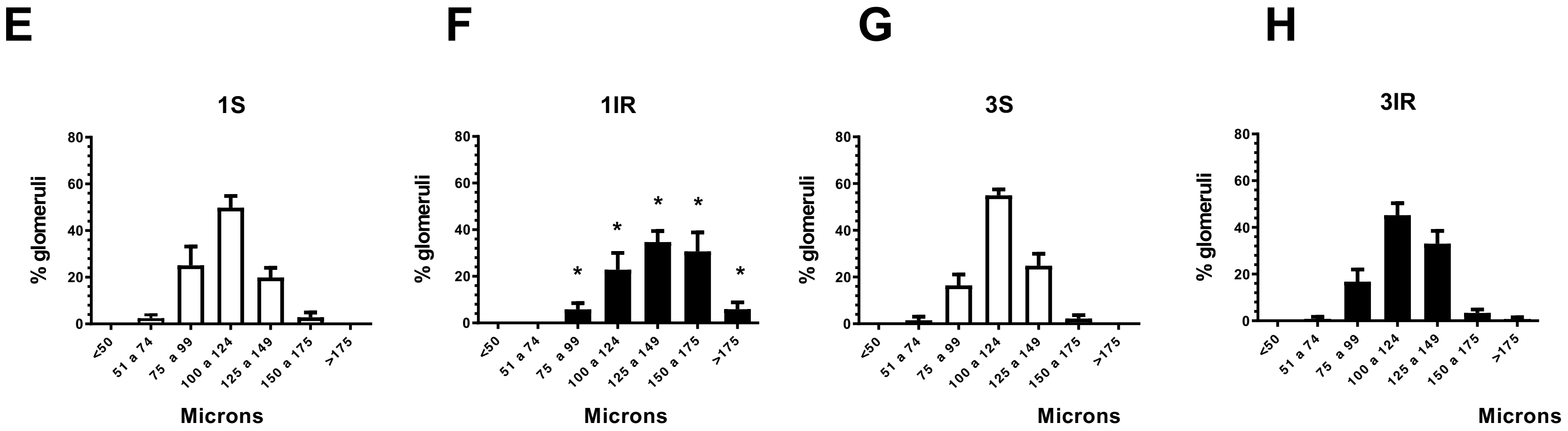

Suppl. Figure S2. Repeated episodes of IR prevent glomerular hypertrophy. Glomerular diameter distribution distributed in seven ranks. The 1S and 3S groups are represented in white bars and the 1IR and 3IR groups in black bars. In the upper panel from A to D are the mild ischemic groups (20 min) and in the upper panel from E to H are the severe ischemic groups (45 min). * $p < 0.05$ vs. its respective S group.

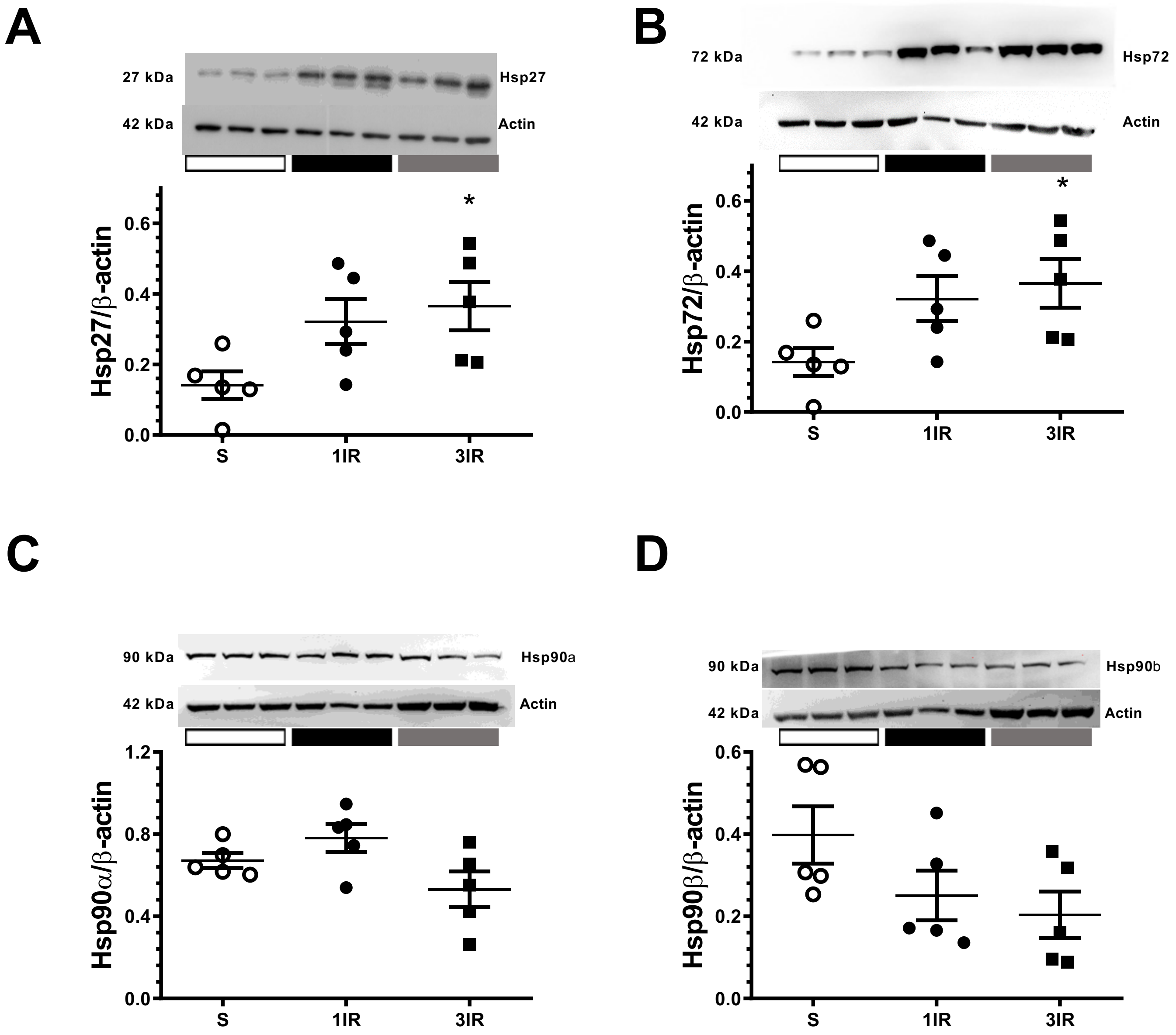

Suppl. Figure S3. Expression of heat shock proteins. Protein expression of heat shock proteins 24 h after the only or last IR episode. A) Hsp27, B) Hsp72, C) Hsp90α and D) Hsp90β. The sham group is represented with white circles, 1IR group in black circles and 3IR group in black rectangles. * $p < 0.05$ vs. S.
